# Supplementary material for: Missense variants in human ACE2 strongly affect binding to SARS-CoV-2 Spike providing a mechanism for ACE2 mediated genetic risk in Covid-19: A case study in affinity predictions of interface variants
Source: PLoS Comput Biol. 2022 Mar 2;18(3):e1009922. doi: 10.1371/journal.pcbi.1009922 (PMC8920257; doi:10.1371/journal.pcbi.1009922)
Supplement: S3 Table — (PDF) [file pcbi.1009922.s003.pdf]

*S3 Table. Association between a binary affinity classification based on predicted ΔΔG with high- and low- binding RBD binding ACE2 variants identified by deep mutagenesis.*

| mCSM-PPI2 (kcal mol$^{-1}$) | nCoV-S High sorts $log_2$ enrichment ratio | | recal. mCSM-PPI2 (kcal mol$^{-1}$) | nCoV-S High sorts $log_2$ enrichment ratio | |
|---|---|---|---|---|---|
| | < 0 | >= 0 | | < 0 | >= 0 |
| < 0 | 304 | 72 | < 0 | 235 | 41 |
| >= 0 | 47 | 14 | >= 0 | 116 | 45 |
| | $\chi^2$ = 0.27, p = 0.6 | | | $\chi^2$ = 10, p = 0.001 | |
