# Supplementary material for: Missense variants in human ACE2 strongly affect binding to SARS-CoV-2 Spike providing a mechanism for ACE2 mediated genetic risk in Covid-19: A case study in affinity predictions of interface variants
Source: PLoS Comput Biol. 2022 Mar 2;18(3):e1009922. doi: 10.1371/journal.pcbi.1009922 (PMC8920257; doi:10.1371/journal.pcbi.1009922)
Supplement: S4 Table — (PDF) [file pcbi.1009922.s004.pdf]

S4 Table. Association between a 4-level affinity classification based on predicted ΔΔG with high- and low- binding RBD binding ACE2 variants identified by deep mutagenesis.

mCSM-PPI2 (kcal mol^{-1})	nCoV-S High sorts log_2 enrichment ratio		recal. mCSM-PPI2 (kcal mol^{-1})	nCoV-S High sorts log_2 enrichment ratio	
	< 0	>= 0		< 0	>= 0
< −1	105	5	< −1	108	5
(−1, 0]	199	67	(−1, 0]	127	36
(0, 1]	45	12	(0, 1]	106	40
>= 1	2	2	>= 1	10	5
	χ^2 = 23, p = 0.00003			χ^2 = 24, p = 0.00002	
