# Supplementary material for: Missense variants in human ACE2 strongly affect binding to SARS-CoV-2 Spike providing a mechanism for ACE2 mediated genetic risk in Covid-19: A case study in affinity predictions of interface variants
Source: PLoS Comput Biol. 2022 Mar 2;18(3):e1009922. doi: 10.1371/journal.pcbi.1009922 (PMC8920257; doi:10.1371/journal.pcbi.1009922)
Supplement: S1 Fig — Distribution of mCSM-PPI2[13] predicted ΔΔG from in silico saturation mutagenesis of the ACE2-S interface in PDB 6vw1[11]. A. predicted ΔΔG for 475 mutations across 25 sites on ACE2 corresponding to the 23 residues within 5 Å of SARS-CoV-2 S plus Gly326 and Gly352. B. predicted ΔΔG for the subset of 151 mutations across these sites that are accessible via a single base change of the ACE2 coding sequence. (PDF) [file pcbi.1009922.s005.pdf]

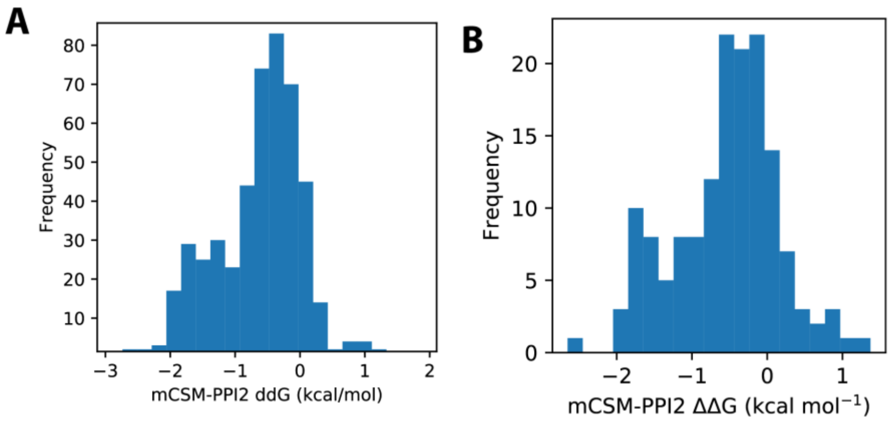

S1 Fig. A. Distribution of mCSM-PPI2[13] predicted ΔΔG from in silico saturation mutagenesis of the ACE2-S interface in PDB 6vw1[11]. A. predicted ΔΔG for 475 mutations across 25 sites on ACE2 corresponding to the 23 residues within 5 Å of SARS-CoV-2 S plus Gly326 and Gly352. B. predicted ΔΔG for the subset of 151 mutations across these sites that are accessible via a single base change of the ACE2 coding sequence.
