# Supplementary material for: Missense variants in human ACE2 strongly affect binding to SARS-CoV-2 Spike providing a mechanism for ACE2 mediated genetic risk in Covid-19: A case study in affinity predictions of interface variants
Source: PLoS Comput Biol. 2022 Mar 2;18(3):e1009922. doi: 10.1371/journal.pcbi.1009922 (PMC8920257; doi:10.1371/journal.pcbi.1009922)
Supplement: S2 Fig — A. Experimental ΔΔG vs. ACE2 mutant enrichment in the high RBD binding population. The regression parameters are: slope = 0.38, intercept = -0.14, R2 = 0.59 and p = 0.01. B. Experimental ΔΔG vs. ACE2 mutant enrichment in the low RBD binding population (negated). The regression parameters are: slope = 0.41, intercept = -0.18, R2 = 0.74 and p = 0.002. Figure generated with R ggplot2. (PDF) [file pcbi.1009922.s006.pdf]

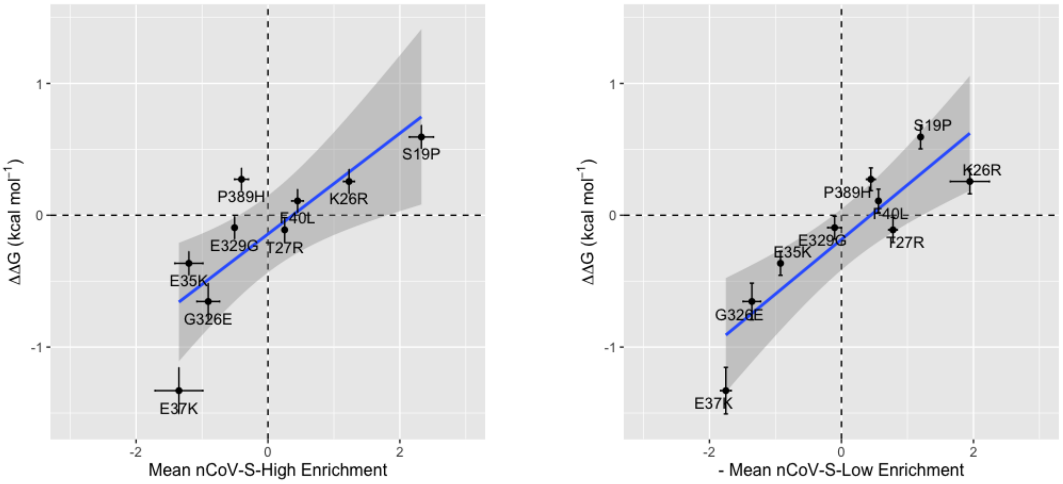

S2 Fig. Comparison of SPR determined ΔΔG with the deep mutagenesis binding data from Procko and co-workers[22]. A. Experimental ΔΔG vs. ACE2 mutant enrichment in the high RBD binding population. The regression parameters are: slope = 0.38, intercept = -0.14, R^2 = 0.59 and p = 0.01. B. Experimental ΔΔG vs. ACE2 mutant enrichment in the low RBD binding population (negated). The regression parameters are: slope = 0.41, intercept = -0.18, R^2 = 0.74 and p = 0.002. Figure generated with R ggplot2.
