# Supplementary material for: Missense variants in human ACE2 strongly affect binding to SARS-CoV-2 Spike providing a mechanism for ACE2 mediated genetic risk in Covid-19: A case study in affinity predictions of interface variants
Source: PLoS Comput Biol. 2022 Mar 2;18(3):e1009922. doi: 10.1371/journal.pcbi.1009922 (PMC8920257; doi:10.1371/journal.pcbi.1009922)

S3 Fig. Protein purification size exclusion chromatography and corresponding SDS-PAGE of labelled peaks. A. WT RBD, B. WT ACE2, C. ACE2 mutants E37K and T27R, (continued next page) D. ACE2 mutants G326E and D355N, E. ACE2 mutants E35K, F40L and S19P, F. ACE2 mutants P329H, E329K and K26R.

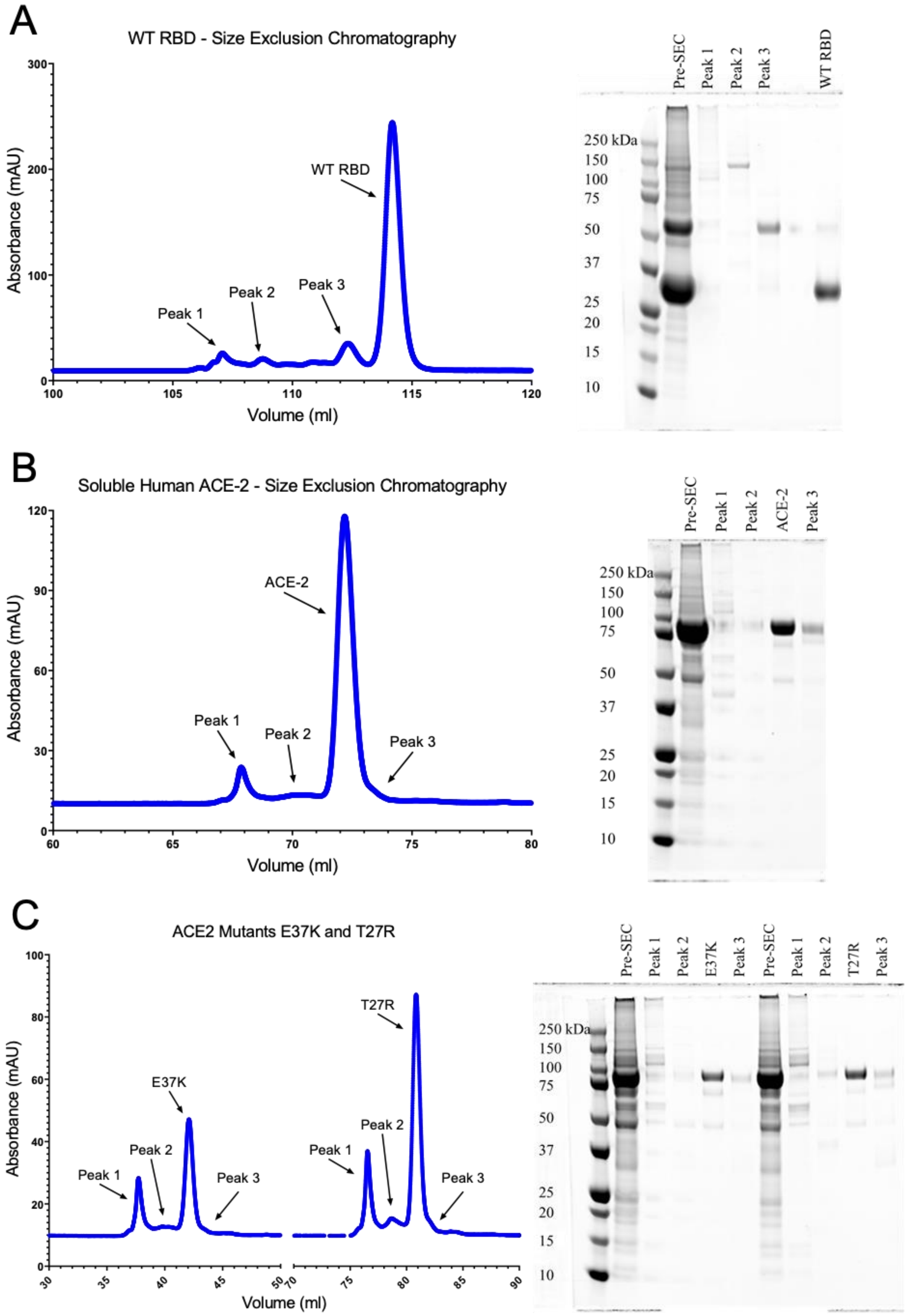

S3 Fig (continued)

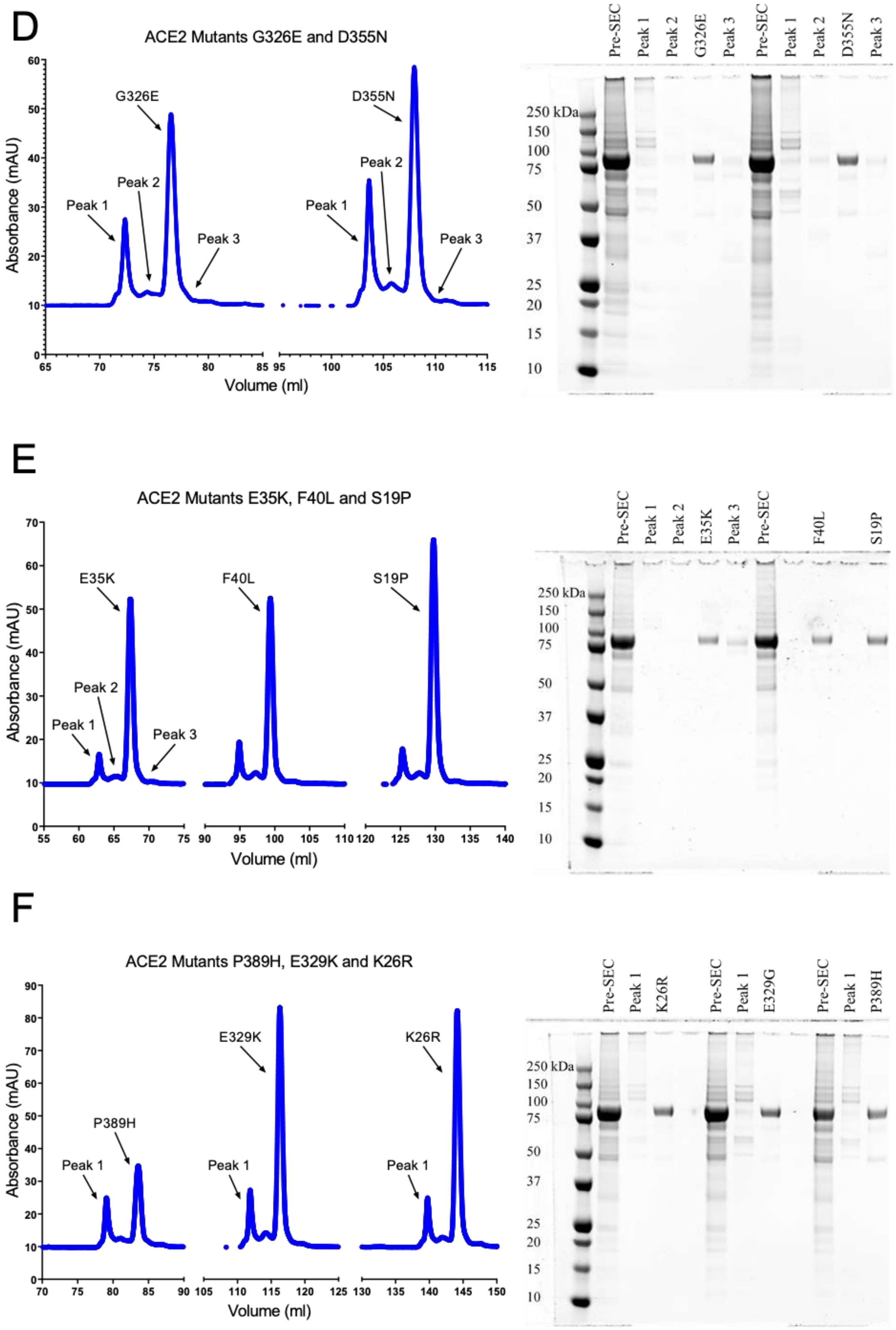

Supplement: S3 Fig — A. WT RBD, B. WT ACE2, C. ACE2 mutants E37K and T27R, (continued next page) D. ACE2 mutants G326E and D355N, E. ACE2 mutants E35K, F40L and S19P, F. ACE2 mutants P329H, E329K and K26R. (PDF) [file pcbi.1009922.s007.pdf]
