# Supplementary material for: Missense variants in human ACE2 strongly affect binding to SARS-CoV-2 Spike providing a mechanism for ACE2 mediated genetic risk in Covid-19: A case study in affinity predictions of interface variants
Source: PLoS Comput Biol. 2022 Mar 2;18(3):e1009922. doi: 10.1371/journal.pcbi.1009922 (PMC8920257; doi:10.1371/journal.pcbi.1009922)
Supplement: S4 Fig — A. Anti ACE2 binding to WT and four ACE2 variants: T27R, G326E, E37K and D355N. B. Binding to WT, S19P and K26R. C. Binding to WT, E35K and F40L. D. Binding to WT, E329G and P389H. Anti ACE2 was injected for 600 s association and buffer for a further 600 s for dissociation. (PDF) [file pcbi.1009922.s008.pdf]

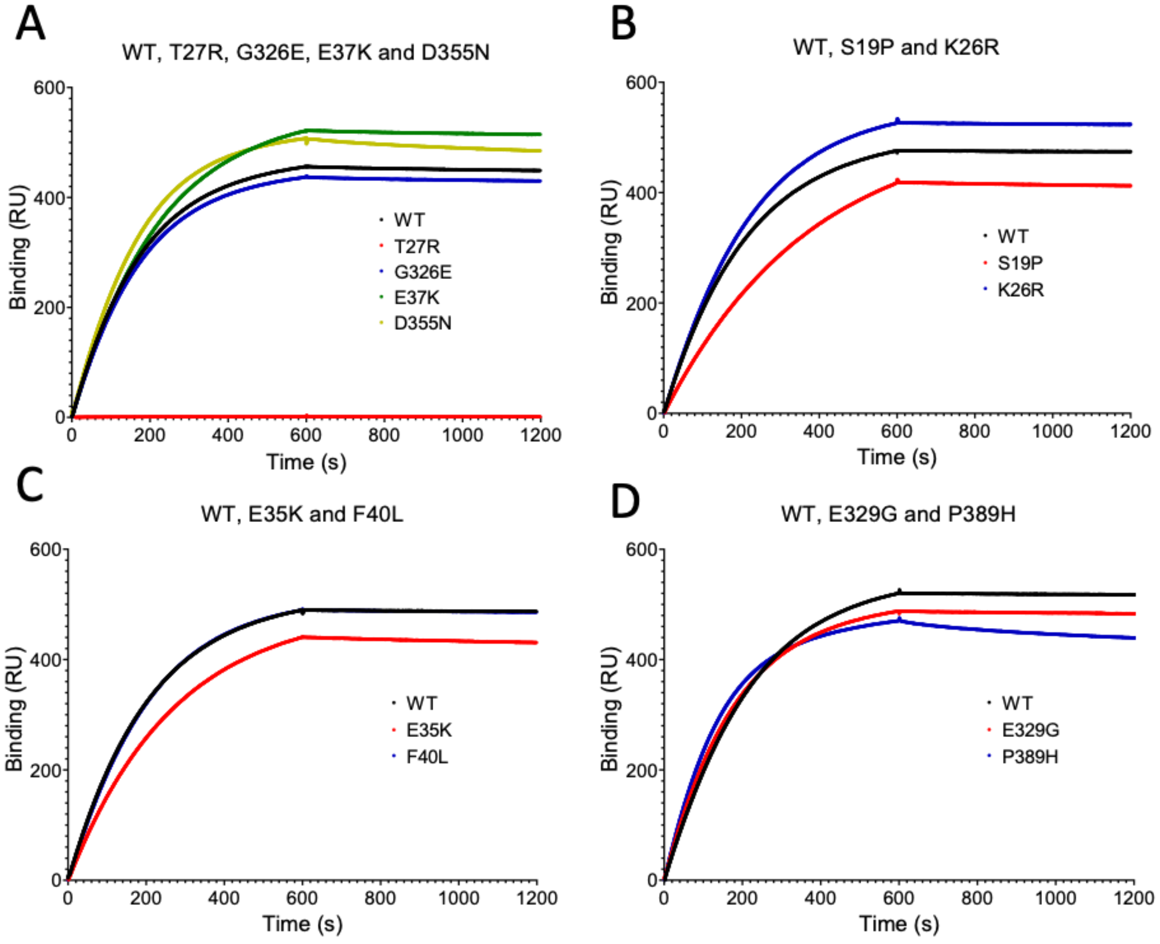

S4 Fig. Anti ACE2 Antibody (NOVUS AC384-NBP2-80038) binding to WT and 10 ACE2 variants. A. Anti ACE2 binding to WT and four ACE2 variants: T27R, G326E, E37K and D355N. B. Binding to WT, S19P and K26R. C. Binding to WT, E35K and F40L. D. Binding to WT, E329G and P389H. Anti ACE2 was injected for 600 s association and buffer for a further 600 s for dissociation.
