# Supplementary material for: Missense variants in human ACE2 strongly affect binding to SARS-CoV-2 Spike providing a mechanism for ACE2 mediated genetic risk in Covid-19: A case study in affinity predictions of interface variants
Source: PLoS Comput Biol. 2022 Mar 2;18(3):e1009922. doi: 10.1371/journal.pcbi.1009922 (PMC8920257; doi:10.1371/journal.pcbi.1009922)
Supplement: S5 Fig — A. Representative binding trace for WT RBD binding immobilised WT ACE2. Injections start from lowest to highest concentration of RBD and fits are shown in blue. B. Plateau binding values from A are plotted against the concentration of RBD injected and the equilibrium KD is extracted from the fit shown in blue. (PDF) [file pcbi.1009922.s009.pdf]

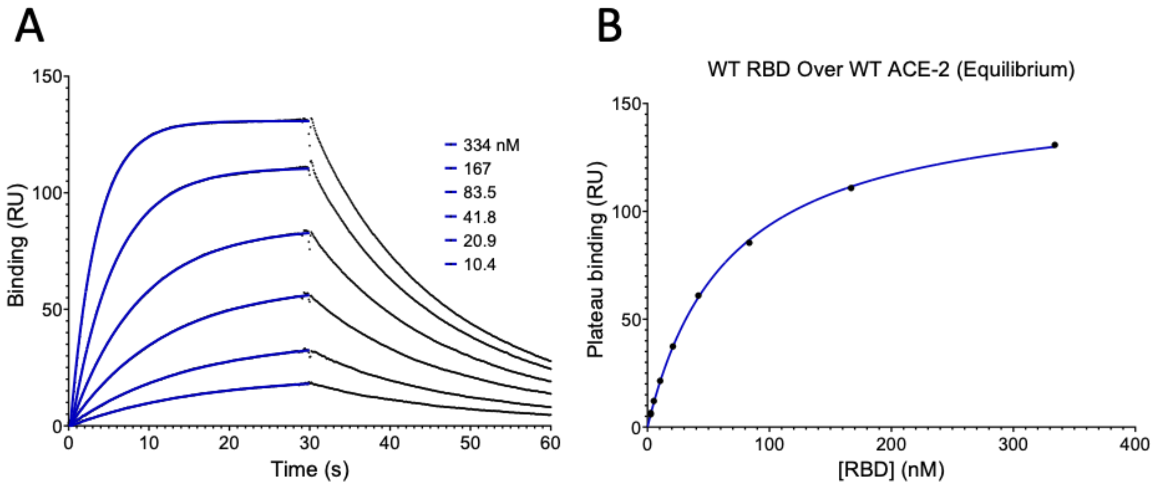

S5 Fig. WT RBD binding WT ACE2 plus equilibrium K_D. A. Representative binding trace for WT RBD binding immobilised WT ACE2. Injections start from lowest to highest concentration of RBD and fits are shown in blue. B. Plateau binding values from A are plotted against the concentration of RBD injected and the equilibrium K_D is extracted from the fit shown in blue.
