# Supplementary material for: Missense variants in human ACE2 strongly affect binding to SARS-CoV-2 Spike providing a mechanism for ACE2 mediated genetic risk in Covid-19: A case study in affinity predictions of interface variants
Source: PLoS Comput Biol. 2022 Mar 2;18(3):e1009922. doi: 10.1371/journal.pcbi.1009922 (PMC8920257; doi:10.1371/journal.pcbi.1009922)
Supplement: Attachment — Submitted filename: ACE2-variants-PLOS-CB-reviewer-response.pdf [file pcbi.1009922.s010.pdf]

Missense variants in human ACE2 strongly affect binding to SARS-CoV-2 Spike providing a mechanism for ACE2 mediated genetic risk and protection from Covid-19

Stuart A. MacGowan[1*], Michael I. Barton[2*], Mikhail Kutuzov[2], Omer Dushek[2], P. Anton van der Merwe[2+] and Geoffrey J. Barton[1+]

Response to reviewers:

Reviewer #1:

In this manuscript the author investigated the contributions of missense variants on ACE2, which affect SARS-CoV-2 binding (through the RBD of the spike protein) on infectivity. First, they use deep sequencing data (created by others) and mCSM-PPI2 to calculate free energy changes of ACE2 interface mutants on binding (this part was partially published by them before in bioRxiv, in a different paper). Next, they chose 10 mutations, with either positive or negative effect on binding, or high occurrence in the population, expressed the ACE2 proteins and measured their binding with the RBD using SPR. The results of the experimental measurements were then used to recalibrate the calculations. They show that the recalibrated calculation had a better fit to the experimental data. From here, they calculated the expected change in binding affinity for all possible interface mutations. Next, they analyzed the single amino acid mutation frequency in the population, using gnomAD. The aim is to see whether carriers of different ACE2 variance will indeed have difference susceptibility to COVID19 (or to severe disease).

1. Figure 5, showing the change in binding free energy before and after recalibration of binding calculations is worrying. After calibration, it seems as if neutral mutations dominate, and the chance of getting higher and lower affinity binding mutations is almost the same. This distribution of effect of mutation on binding is different from what has been found before (see for example https://www.ncbi.nlm.nih.gov/pmc/articles/PMC4492929/). This shed doubt of the general applicability of the calibrated calculation. To test this, one should use additional mutations, not included in the experimental data from this manuscript, to test the validity of the calibrated calculations. Such mutation data are now available in a number of other recently published papers.

This comment highlights a critical aspect of our recalibrated ddG predictions where more mutations were found to have neutral or small positive effects on binding than the uncalibrated predictions and raises two important points: (1) Does this result conflict with expectations given current understanding of the effects of mutations at interfaces? (2) Can we test the recalibrated data further with an external dataset?

The histogram in Figure 6 (p. 12; previously Figure 5) shows that although variants with small predicted ddG are centred around ddG = 0 kcal mol^{-1} following recalibration (cf. Supplementary Figure 1, p. 30 for original mCSM-PPI2 predictions), there is still a secondary mode below −1 kcal mol^{-1} and therefore many more variants are predicted to disrupt binding than enhance binding, as is expected from various reports in the literature. We originally concluded that these features of the recalibrated predictions indicated better performance, supported also by the observation that variants that were erroneously predicted to weakly disrupt or have no effect on binding (i.e., ACE2 S19P and K26R) were correctly classified after recalibration, and the fact that that mCSM-PPI2 was trained on a dataset biased toward lower affinity mutations.

We now have stronger evidence that recalibration provides an improved ddG prediction thanks to the reviewer's suggestion to compare our results to external datasets (p. 9-10). Briefly, we compared the ddG predictions with the deep mutagenesis binding data reported by Procko and co-workers[1] (Figure 5, p. 10; newly added) and our analysis suggests that: 1) ddG < −1 kcal mol^{-1} is a highly specific indicator of significantly decreased affinity with or without recalibration 2) ACE2 mutants that are predicted to cause small changes in affinity showed both increased and decreased affinity in the deep mutagenesis binding data and there is significant discordance between the datasets in this region, suggesting that these mutants are most appropriately distributed around ddG = 0 kcal mol^{-1} as they are after recalibration, and 3) recalibrated ddG is more sensitive towards variants that increase affinity whilst the original ddG seems to overpredict disrupted binding. Further conclusions are derived when we highlight how the deep mutagenesis data are not an ideal gold standard for the predictor because the effects of variable ACE2 expression and these provide further hints that recalibration results in an improved prediction (p. 9-10).

2. As mentioned in the paper, infectivity of pseudotype virus has been investigated for S19P and K26R, for which tighter binding was measured, but no increase in infectivity was observed. It would be helpful for this manuscript to take also the binding data published recently by Shukla (https://doi.org/10.1371/journal.ppat.1009715) and see how they fit into the calculated binding data in this manuscript. Moreover, use the infectivity data of Shukla, to compare to the binding calculation in this manuscript.

It turns out that most of the binding data reported in Shukla et al.[2] was taken from the deep mutagenesis binding dataset reported by Procko and co-workers and so our above response and our comparison of these data to predicted ddG (p. 9-10) addresses the first part of this comment.

As suggested, we compared our affinity results to the pseudotype infectivity data reported in Shukla et al. and have included this analysis in the revised manuscript (p. 13-14). We observed a remarkable correlation between pseudotype infectivity and experimental ddG amongst the six variants that could be compared (Figure 7, p.13; newly added). Predicted ddG was also correlated to these infectivity data and although the association was not as well-defined, the analysis suggests that predicted ddG provides a useful indication of how a variant may alter infectivity.

These new results provide further insight into the relationship between affinity and infectivity and provide substantial experimental evidence for our original argument that

affinity lowering variants are likely to reduce infectivity (p. 12, para. 1). Shukla et al's results regarding the interplay between variable expression levels and RBD affinity of ACE2 mutants also support our hypothesis that RBD affinity enhancing ACE2 variants may result in a broader susceptibility of different host cell-types to infection (p. 12-14).

3. Shukla adds another interesting point, that the surface abundance of ACE2, which is affected by the variant, is of major importance in infectivity. Please discuss this point in the manuscript.

We agree this is an important point, and it is now discussed at greater length in the revised manuscript (pp. 10, 12-14; see also response above). What is particularly interesting is the interplay between RBD affinity and ACE2 surface abundance that was discovered. These findings are consistent with the relationship between receptor affinity, surface density and infectivity in other enveloped viruses[3], which underpinned our hypothesised mechanism for the potential of affinity increasing mutants to lead to increased infectiousness or disease severity in the host (p. 12, para. 2).

4. A number of variants of SARS-CoV-2 are now circulating and dominating infection (the Wuhan strain is rare now). Using binding calculations to the alpha, beta and delta variants, to ACE2 variants could provide further interesting information of the relation between potential infectivity and variants in ACE2 and SARS-CoV-2.

The effects of ACE2 variants on RBD affinity can differ between the different Spike variants and in a related paper that focussed on RBD variants we showed that there was a particularly strong antagonism between ACE2 S19P and Spike S477N[4]. Additionally, Shukla et al also showed that the Spike N501Y variant rescues infectivity towards ACE2 K353D, D355N and D38H[2], which presumably is in part due to compensation of the reduced affinity of these ACE2 variants by the increased affinity of Spike N501Y. Interestingly, the infectivity of ACE2 R357A, E37K and Y41A were not increased by Spike N501Y[2], which we think indicates a non-additive relationship between the affinities of these ACE2 variants and Spike N501Y. We have commented on these points in the revised manuscript (p. 15).

We agree that predictions will be extremely useful to explore the relationship between variants in ACE2 and Spike RBD to monitor variants for potential genotype specific risks, particularly with the common ACE2 variant alleles S19P and K26R. Unfortunately, these calculations are beyond the scope of the present work as it will take substantial effort to obtain and calculate these predictions, which in some cases will require modelling multiple mutations on Spike RBD alongside the variants in ACE2 and further experimental efforts to validate the results. We have highlighted this avenue of future research in our revised conclusion (p. 18).

5. In figure 4 show both the correlation of the original and recalibrated calculation to the experimental data.

We have added an extra panel to Figure 4 comparing the recalibrated calculation to the experimental data as requested (p. 9).

6. Why are recalibrated calculation data missing for T27R and G326E? these were the most interesting points in the original calculations.

Unfortunately, these high predicted ddG values proved to be erroneous according to our RBD binding experiments and so in these specific cases we knew that the recalibration didn't improve the prediction because they were not correlated with the experimental data. Indeed, for these variants recalibrated scores have even poorer agreement with experiment. Our initial conclusion was to consider high positive predicted ddG as unreliable (p. 8) and this was why we deemed recalibration as not applicable for these variants However, the new analysis comparing the deep mutagenesis binding assay to the ddG predictions (pp. 9-10) indicates that recalibration is useful for positive predicted ddG even though they are probably less reliable than strongly negative predicted ddG, which all evidence suggests are extremely accurate.

We have emphasized the problems we see with high affinity increasing predictions (especially T27R and G326E) and our conclusion that affinity enhancing predictions are less reliable than affinity lowering predictions throughout the largely rewritten and expanded §2.4 (p. 8-10). We have also provided the recalibrated ddG values in Table 1 with a footnote that explains these caveats (p. 5).

Reviewer #2

The manuscript represents an interesting analysis of mutations in ACE2, the receptor used by the SARS-CoV-2 spike protein to enter the host cell. The specific focus is on variation with the human ACE2 gene that occurs most frequently within the large gnomAD population. The research uses the binding data from SPR experiments to recalibrate the mCSM-PPI2 specifically to predict the effect of other ACE2 variants that have not been studied by SPR. This highlights a good use of wet laboratory data to improve the accuracy of predictions to provide greater insight into variants within a specific protein.

1. The manuscript refers to previous in silico research performed. This is reference 23, which is listed as a bioRxiv preprint. It seems unusual to refer to highly related work by the same researchers that is in a preprint. Is that research under consideration for publication elsewhere? Alternatively, I wonder if it should be presented in this manuscript. I am not sure what to make of this, we are clearly in a time when there is increasing use of preprints and this is certainly to be encouraged. However, in this case it does not aid readability of the manuscript.

There is some overlap between this submission and the referred bioRxiv preprint but there are significant differences in the methods, results, conclusions, and authors that merit each as distinct papers. The preprint refers to the efforts of MacGowan and Barton to assess the effects of ACE2 variants on Spike RBD binding using the mCSM-PPI2 predictor alone and to contextualise these results by benchmarking the predictor's performance on previously published binding data from the mid-2000s for ACE2 in complex with SARS-CoV-1 Spike. Since we did not have the experimental SPR data reported in the current submission, we also relied heavily on a structural analysis to rationalise the predictions of mCSM-PPI2. In contrast, in this work the primary results are the experimental binding affinities for the gnomAD variants, including the major finding that two common variants enhance affinity (S19P and K26R; which in our earlier preprint we thought did not significantly alter binding)

as well as the recalibrated mCSM-PPI2 predictions, which are used in the prediction of total burden of rare variants that effect RBD binding.

It is important to highlight that the preprint is not presently under consideration and nor will we submit this preprint for publication in future as this PLOS CB submission makes it largely obsolete. However, it is important that the preprint remains visible as it has been cited by others, preserves the timeline of our work (we made those results public in May 2020) and we choose to cite this work ourselves where appropriate rather than incorporating further results and methods into this submission as this would result in a complicated and long paper, without necessarily adding to the main scientific arguments.

2. The text referring to GWAS of ACE2 variants, could be clearer, readers may not be familiar with beta etc.

We have added explanations of the GWAS parameters that are cited to the caption of Table 2 and cross-referenced these definitions in the text and we have also simplified the table by removing less relevant rows and columns.

3. The main text would benefit from a brief comment about the mCSM-PPI2 recalibration. I appreciate that this is covered in the methods but it would likely improve readability.

We have substantially increased the depth of our description of the recalibrated predictions in the revised manuscript in response to the other reviewer's comments (p.8-11) and we have specifically included a brief description of how the recalibrated results were calculated within the main text as requested here.

4. The paragraph that starts with "Figure 4" is very long and difficult to read. The conclusion is also one long paragraph.

We have rewritten the paragraph starting "Figure 4…" to focus on the key argument for the applicability of recalibration (p. 8).

We have significantly expanded the conclusion to more completely summarise the main findings, applications and broader impact of our work.

5. Is the following statement in the abstract relating to use of SPR appropriate or needed? "37ÅãC, taking care to avoid common pitfalls with this method"

Although our experimental protocol was carefully conducted to avoid artefacts from protein aggregation, mass-transport limitations, and rebinding, we aggree this comment can be removed from the abstract for the sake of brevity.

Other minor changes

- Updated abstract to include affinity vs. infectivity analysis
- Updated Biorxiv reference to published version
- Added missing footnotes to Table 1

- Renamed §2 "Results and Discussion" (formerly "Results") and §3 "Conclusion" (formerly "Conclusion and Discussion") and placed the sections exploring the relationship between affinity and infectivity and carrier vulnerability (§2.6; formerly §3.1) and the total prevalence of ACE2 alleles with altered Spike affinity (§2.7; formerly §3.2) at the end of §2. This fits better now that we compare the RBD affinities of the ACE2 variants to Shukla et al's pseudotype infectivity data.
- Some minor editing throughout the manuscript to improve clarity. All changes have been tracked.

References

1 Chan, K. K. *et al.* Engineering human ACE2 to optimize binding to the spike protein of SARS coronavirus 2. *Science* **369**, 1261-1265, doi:10.1126/science.abc0870 (2020).

2 Shukla, N., Roelle, S. M., Suzart, V. G., Bruchez, A. M. & Matreyek, K. A. Mutants of human ACE2 differentially promote SARS-CoV and SARS-CoV-2 spike mediated infection. *PLoS Pathog* **17**, e1009715, doi:10.1371/journal.ppat.1009715 (2021).

3 Hasegawa, K. *et al.* Affinity thresholds for membrane fusion triggering by viral glycoproteins. *J Virol* **81**, 13149-13157, doi:10.1128/JVI.01415-07 (2007).

4 Barton, M. I. *et al.* Effects of common mutations in the SARS-CoV-2 Spike RBD and its ligand, the human ACE2 receptor on binding affinity and kinetics. *Elife* **10**, doi:10.7554/eLife.70658 (2021).
