# Supplementary material for: Missense variants in human ACE2 strongly affect binding to SARS-CoV-2 Spike providing a mechanism for ACE2 mediated genetic risk in Covid-19: A case study in affinity predictions of interface variants
Source: PLoS Comput Biol. 2022 Mar 2;18(3):e1009922. doi: 10.1371/journal.pcbi.1009922 (PMC8920257; doi:10.1371/journal.pcbi.1009922)
Supplement: Attachment — Submitted filename: Response_to_editor_PostAppeal.docx [file pcbi.1009922.s011.docx]

Dear Charlotte and Arne,

Thank you for looking again at our manuscript. In response to your suggestions and the referee’s comments we have made some changes to clarify the fit on Figure 4 and also the text around Figures 5 and 7. We detail the changes below and in the track changed copy of the manuscript. These include a change to the Title and Abstract to alert readers to the general relevance of our work beyond the current pandemic.

We hope that these changes help clarify the message and satisfy the earlier concerns about the manuscript.

With best regards,


Geoff.

**Changes**

1. Title and abstract now reflect both the relevance to Covid-19 and the wider issue of interpreting affinity predictions for interface variants

2. Page 8 and Legend to Figure 4. We have added additional clarification about the fitting shown in Figure 4, the reasons for this and how we use the fit to help with interpretation of the mCSM-PPI2 predictions.

3. Page 9-10 and Figure and legend to Figure 5. We have updated Figure 5 to include dotted lines at +/- 1 kcal mol-1 to help guide the reader’s eye. We have also extended the text to clarify further how the mCSM-PPI2 and our recalibrated values fit to the enrichment data.

4. Table 3. We have added a footnote to this table to emphasise the caveats around mCSM-PPI2 predictions of +ve ddG.

5. Figure 7 legend and associated text on page 14 has been extended further to clarify the agreement with pseudovirus infection data and how the mCSM-PPI2 recalibrated predictions perform better than published comparisons to the Procko deep mutagenesis data.

6. We have added a sentence at the end of Conclusions on Page 18 to emphasise the general importance of the work beyond COVID-19 and its limitations.
